# Targeted-Produced Dirhamnolipids from *Pseudomonas aeruginosa* Induce Antinociception in Mice

**DOI:** 10.1021/acsomega.5c03648

**Published:** 2025-08-07

**Authors:** Kamila B. B. Wessel, Ana Paula Mello, Ismael Rodrigues Amador, Marília F. Manchope, Nayara Rampazzo Morelli, Anelise Franciosi, Tiago H. Zaninelli, Mariana M. Bertozzi, Cesar A. Tischer, Renata M. Martinez, Nicole Caldas Pan, Marcela M. Baracat, Rubia Casagrande, Waldiceu A. Verri, Doumit Camilios-Neto, Josiane A. Vignoli

**Affiliations:** 1 Departamento de Bioquímica e Biotecnologia, Centro de Ciências Exatas, 37894 Universidade Estadual de Londrina, Londrina 86057-970, Brazil; 2 Departamento de Imunologia, Parasitologia e Patologia Geral, Centro de Ciências Biológicas, 37894 Universidade Estadual de Londrina, Londrina 86057-970, Brazil; 3 Departamento de Ciências Farmacêuticas, Centro de Ciências da Saúde, 37894 Universidade Estadual de Londrina, Londrina 86057-970, Brazil

## Abstract

Rhamnolipids are highly effective surface-active glycolipid biosurfactants with enormous market potential. Composed of β-d-(β-d-hydroxyalkanoyloxy)­alkanoic acids attached to mono- or dirhamnose moieties, these green amphiphilic molecules exhibit remarkable biomedical prospects, with the double rhamnose congeners being the most effective ones. Although reports on dirhamnolipid antimicrobial activities and wound healing properties are quite abundant, the antinociceptive effect on inflammatory pain has never been tested before. Here we report a targeted-producing dirhamnolipid process, which reaches 95.1% of dirhamnolipid abundance, followed by a single-step purification procedure through a homemade silica cartridge, achieving highly pure glycolipid (99.0% rhamnolipids and 97.5% dirhamnolipids). Purified Di-RL (pDi-RL) therapeutic effects were investigated in murine models of pain and inflammation induced by carrageenan, acetic acid, and formalin. Mice were pretreated with pDi-RL at doses of 0.3 and 3 mg/kg, subcutaneously, 30 min before the inflammatory stimulation. pDi-RL at the 3 mg/kg dose reduced the mechanical sensitivity and leukocyte infiltrate in the cutaneous plantar skin induced by carrageenan and overt pain-like behaviors induced by formalin and acetic acid. Furthermore, the pDi-RL mechanisms were assessed in a peritonitis model induced by carrageenan, and pDi-RL reduced the total leukocyte recruitment (mononuclear and polymorphonuclear cells) and superoxide anion production by recruiting leukocytes in the peritoneal exudate. Here, we demonstrate for the first time the antinociceptive activity of Di-RL and, at least in part, its mechanism in murine models of pain and inflammation.

## Graphical abstract

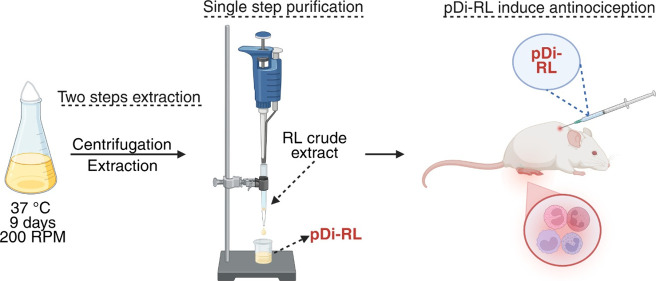

## Introduction

Inflammation is a physiological and protective body response that occurs to combat tissue damage and/or an infection aiming to restore tissue homeostasis.^1^ Tissue-resident macrophages and mast cells recognize specific molecular structures in microorganisms or danger signals released during sterile inflammation through pattern recognition receptors (PRRs), detecting signals derived from pathogens or damaged tissue.^2,3^ In acute inflammation, the recruited leukocytes are monocytes and mainly neutrophils, which produce reactive oxygen species (ROS), such as superoxide anion and nitric oxide.^4,5^ The superoxide anion is a central ROS since it gives rise to other ROS, such as hydroxyl radical, hypochlorous acid, and singlet oxygen,^6^ which lead to tissue damage triggered by oxidative stress.^7^ However, superoxide anion is produced not only upon the arrival of recruited leukocytes in the inflammatory primary foci, but rather, this ROS also participates in the recruitment of leukocytes such as the neutrophil. By inhibiting NADPH oxidase, which is an enzyme that produces superoxide anion and is expressed by neutrophils, there is a loss in the directionality toward the chemoattractant, and multiple pseudopods are formed, resulting in reduced chemotaxis.^8^ Neutrophils also express the enzyme myeloperoxidase, which uses the superoxide anion itself together with a halite, such as Cl, to produce hypochlorous acid. This molecule plays a protective role against pathogens but can also contribute to tissue damage when combined with superoxide anion.^9^

Quite interestingly, neutrophils contribute to pain as well as superoxide anion production.^10–12^ Pain is an unpleasant experience that involves a sensory dimension of the detection and neurotransmission of nociceptive stimuli. It also involves an emotional dimension related to prior live experiences and the individual’s response to painful stimuli. Both dimensions are triggered by tissue damage or even an experience resembling that of tissue lesion.^13^ Pain is one of the cardinal inflammation signs and a clinical symptom that is decisive in searching for treatment.^14^ Inflammatory pain occurs by the sensitization of primary sensory neurons (nociceptors) with inflammatory mediators^15^ and can be triggered by oxidative stress and free radicals since superoxide anion injection induces nociceptive behaviors in mice^16–18^ and activates nociceptive neurons.^19^ The superoxide anion donor potassium superoxide injected in the paw induces paw flinches and licking for 30 min, and in the peritoneum, the injection caused a writhing behavior in mice. Moreover, the superoxide anion donor increases mechanical and thermal hyperalgesia accompanied by paw edema and neutrophil recruitment in mice.^16^

The treatment of inflammatory pain relies mainly on nonsteroidal anti-inflammatory drugs (NSAIDs), which act by inhibiting prostaglandin-endoperoxide synthase enzymes (e.g., cyclooxygenase-1 and -2), responsible for the synthesis of prostaglandins and well-known for their role in inflammatory pain. However, the use of this class of drugs can cause significant adverse effects, including gastric mucosal injuries and kidney damage.^20^ Therefore, it is of great importance to develop novel drugs that control pain and inflammation and do not cause such side effects.^21^

Rhamnolipids (RLs) are glycolipids consisting of one or two rhamnose molecules, respectively called mono- and dirhamnolipids (Di-RLs), linked to β-hydroxylated fatty acids.^22,23^ These compounds are biosurfactants mostly produced by the bacterium *Pseudomonas aeruginosa* as a mixture of congeners, being varieties of mono- and Di-RL.^24^ RLs have known antibacterial^25–28^ and antifungal^25,28,29^ activities and are widely used in agriculture, bioremediation, food, cosmetic, pharmaceutical, and cleaning product industries.^23,28^ They are the most extensively studied biosurfactants due to their high production yields, excellent physicochemical properties, biodegradability, and remarkable stability under extreme conditions.^22,30^ RLs are cytotoxic *in vitro* against some types of human cancer cells^28^ [leukemic Hl-60 (acute myeloid leukemia), BV-173 (chronic myeloid leukemia in blast crisis), SKW-3 (T-cell lymphocytic leukemia),^28,31^ human breast cancer cell line (MCF-7),^32^ and bladder cancer cells].^31^ Additionally, Di-RLs induce wound healing in animal models.^28^ Topical treatment with Di-RL ointment (0.1% in Eucerin) accelerates the healing of burn wounds induced by boiling water in Sprague–Dawley rats by reducing collagen fibers in the injured area.^33^ On a rabbit ear, the topical treatment of Di-RL (2 g/L in PBS) inhibited keloid formation caused by puncture; this effect occurs via a cell death mechanism directed at myofibroblasts.^34^ Topical treatment of Di-RL (ointment, 5 g/L) has effects on the healing of 8 mm circular blade excised wounds in Wistar rats due in part to its antimicrobial action against *Staphylococcus aureus* ATCC6588.^35^ Di-RL has low toxicity, and its use is safe, being approved by the Food and Drug Administration for use in vegetable, fruit, and legume crops.^36^ In the present study, we sought to purify Di-RL produced by *P. aeruginosa* and test its antinociceptive and anti-inflammatory effects in murine models of inflammatory pain.

## Materials and Methods

### Production and Extraction of Rhamnolipids

*Pseudomonas aeruginosa* PAO1 was the strain used for RL production.^37^ PAO1 was stored in a Luria–Bertani (LB) broth with 20% glycerol at −80 °C. Seed culture was prepared by inoculating colonies from the LB agar plate into 25 mL of the LB broth within a 125 mL Erlenmeyer flask. The culture was incubated at 37 °C under agitation in an orbital shaker (200 rpm) until the midexponential phase (optical density at 600 nm between 0.6 to 0.8). Two milliliters of the midexponential phase seed culture was inoculated into a 250 mL Erlenmeyer flask containing 100 mL sterile salt solution, contained, per liter, 3.0 g KH_2_PO_4_, 7.0 g K_2_HPO_4_, 0.2 g MgSO_4_·7H_2_O, 1 g (NH_4_)_2_SO_4_, and 3% (v/v) glycerol. Submerged cultivations were incubated in an orbital shaker at 200 rpm for 9 days at 37 °C. Cultures were interrupted by centrifugation at 2500*g* for 20 min.^38,39^ The cell-free supernatant was acidified to pH 2 with 1 M HCl solution and stored at 4 °C for 5 days followed by 2500*g*/25 min/4 °C centrifugation. The RL pellet was extracted with chloroform–methanol (9:1, v/v). The organic solvents were evaporated through reduced pressure at 60 °C, giving rise to a crude extract of RL, which was solubilized in distilled water and lyophilized.

### Rhamnolipid Purification

A silica cartridge purification procedure was carried out as previously described.^40^ Briefly, 300 mg of silica gel 60 PF254 (Merck) was placed in a 5 mL cartridge tip packed and activated with 5 mL of methanol followed by a cleaning step with 5 mL of dichloromethane. The lyophilized crude RL was solubilized in methanol and applied directly to the silica cartridge followed by two steps of cleaning with 5 mL of dichloromethane and 5 mL of chloroform/2% methanol. Purified Di-RL (pDi-RL) was recovered by elution of 5 mL methanol/2% acetic acid.^40^ pDi-RL was evaluated by orcinol-stained thin layer chromatography (TLC) on silica (DC-Fertigfolien ALUGRAM Xtra SIL G/UV254, 20 × 20 cm) with a mobile phase of chloroform/methanol/acetic acid (65:15:2). pDi-RL was also subjected to mass spectrometry analysis and liquid nuclear magnetic resonance. The first was performed on a high-resolution Impact II mass spectrometer (Bruker Daltonics Corporation, Germany) with Q-TOF geometry equipped with an electrospray type ionization source and operated in negative mode with an acquisition rate of 1 Hz (MS and MS/MS) in the mass range of *m*/*z* 50–600. The analyses were performed with a capillary voltage of 4.0 kV, source temperature of 180 °C, and desolvation gas flow of 4 L·min^–1^. MS/MS experiments were performed using collision-induced dissociation (CID) with a collision energy ramp in the range of 15–40 eV.

### Animals

Female Swiss mice used (20–25 g) were from Londrina State University, Londrina, Paraná, Brazil. Mice were kept in standard clear cages with water and food *ad libitum* in a light/dark cycle (12/12 h) and controlled temperature (23 °C). The behavior experiments were carried out between 9:00 a.m. and 5:00 p.m. in a temperature- and sound-controlled room. Animals were euthanized by 5% isoflurane inhalation followed by exsanguination. This study was approved by the Animal Welfare Department of Londrina State University (CEUA-UEL: 037.2020). All efforts were made to minimize animal numbers and suffering.

### Reagents and Drugs

The compounds used in this study were saline (NaCl 0.9%, Eurofarma Laboratórios S.A., Ribeirão Preto, SP, Brazil), carrageenan (Santa Cruz Biotechnology, Santa Cruz, CA, USA), nitroblue tetrazolium dye (NBT), hematoxylin and eosin (Laborclin, Pinhais, PR, Brazil), xylol (Synth, Diadema, SP, Brazil), acetic acid and formaldehyde (Mallinckrodt Baker, S.A., Mexico, Mexico City), and pDi-RL obtained as aforementioned.

### Experimental Procedures

Mice were treated subcutaneously (sc) with pDi-RL (0.3 and 3 mg/kg) or vehicle (saline 0.9%) 30 min before carrageenan stimulation by intraperitoneal (ip; 1 mg, 100 μL) or intraplantar (ipl; 300 μg, 20 μL) routes. The saline group was used as a negative control of carrageenan. Mechanical hyperalgesia was assessed using von Frey filaments or their electronic version at 1, 3, and 5 h after carrageenan stimulus. The dose of 3 mg/kg of Di-RL was chosen for the following experiments. Overt pain-like behavior was evaluated through paw flinches induced by formalin (1.5% formaldehyde, 20 μL, ipl) or writhings induced by acetic acid (0.8% acetic acid, 10 mL/kg, ip). The number of paw flinches was assessed over 30 min after administration, and writhings were observed during 20 min after stimulus. Paw tissue was collected after 5 h of carrageenan-induced inflammatory pain for histopathology analysis. Total leukocyte recruitment and polymorphonuclear and mononuclear cells, as well as superoxide anion-positive leukocytes, were assessed in the peritoneal lavage 5 h after carrageenan-induced peritonitis.

### Mechanical Hyperalgesia Test

The sensitivity to mechanical stimulation was assessed by the electronic von Frey method.^41^ This test consists of applying a mechanical stimulus to the animals’ hind paw with a 0.5 mm^2^ diameter tip. Thus, increasing pressure was applied to the right hind paw until the pressure required for the paw withdrawal response was recorded, and the force (g) required to induce this nociceptive response was quantified by a digital analgesimeter (Insight). The results were expressed by the delta value (Δ) obtained by the difference between the measurement of each animal on the time points 1, 3, and 5 h after the stimulus with carrageenan (ipl; 300 μg, 20 μL) and the measurement before the stimulus (baseline).

To further measure the mechanical sensitivity, the von Frey filament test (Aesthesio) was also performed, as described by Chaplan et al. (1994).^42^ The mechanical stimulus was applied to the animal’s right hind paw using a set of filaments of von Frey with increasing bending forces (0.07, 0.16, 0.4, 0.6, 1.0, 1.4, 2.0, and 4.0 g). Each filament was kept in the paw for 2 to 3 s, being slightly flexed, and the pressure caused by a given filament was detected by the movement of withdrawal of the paw. In the absence of a paw withdrawal response to the initially selected filament, a thicker filament corresponding to a stronger stimulus was used. In the case of paw withdrawal, a weaker stimulus was used. The analysis of the result was carried out by a computer program, the UDReader (Up–Down Reader), as described previously by Gonzalez-Cano et al. (2018).^43^ The results were expressed by the log of gross values.

### Overt Pain-like Behaviors

We used two models of overt pain-like behavior. Writhing was induced by an ip stimulus with acetic acid (0.8% acetic acid). Each mouse was individually placed in a glass cylinder, and the writhing behavior was assessed for 20 min poststimulation. Results are expressed by the total number of writhes over 20 min.^44^ For the formalin test, mice were stimulated ipl with formalin solution (1.5% formaldehyde), which induced a biphasic response over 30 min. The neurogenic phase was observed for 0–5 min, and the inflammatory phase was between 10 and 30 min after the formalin stimulus. The number of paw flinches was counted during both phases. Results were indicated by the number of flinches in both phases.^45^

### Histopathology

Five hours after carrageenan-induced inflammatory pain, cutaneous plantar tissue was collected for histopathological analysis. The samples were fixed with 10% formalin for 24 h and then washed in running tap water for 2 h. Subsequently, plantar cutaneous tissue was dehydrated in ethanol baths with increasing concentrations (70, 80, 95, and 100%) followed by diaphanization with xylol for paraffin embedment. Six slices of each sample were cut in a serial interrupted manner. Slides were stained with hematoxylin–eosin (H&E) and imaged by optical microscopy at 40× objective (Olympus Life Science, model C×31RTSF, Tokyo, Japan). The score parameters were (a) inflammatory infiltrate (1 = no inflammation, 2 = mild, 3 = moderate, and 4 = severe inflammation) and (b) and vascular proliferation (1 = no vascular proliferation, 1 = mild, 3 = moderate, and 4 = severe vascular proliferation). The final score was determined by summing the two parameters. The experimenter was blinded to the experimental groups. Images were analyzed using the ImageJ software (developed at the U.S. National Institutes of Health and available on the Internet)^46^ to assess the leukocyte infiltration through pixels, given in percentage of nucleus staining per dermal area.

### Peritonitis

Peritonitis was induced by carrageenan (1 mg per peritoneal cavity) ip stimulation for 5 h. The peritoneal cavity was washed using 1 mL of 2 mM EDTA containing 0.5% of BSA. The total leukocyte number was counted in a Neubauer chamber using Turk’s solution to lyse red blood cells (10 μL of lavage plus 180 μL Turk’s solution). Differential counting was performed in a slide stained with the Romanowsky stain-based method (Fast Panoptic Kit, Laborclin, Pinhais, PR, Brazil). The results were presented as the number of total leukocytes and polymorphonuclear and mononuclear cells × 10^6^.^47,48^

### Nitroblue Tetrazolium (NBT) Assay

Peritoneal lavage was collected 5 h after the carrageenan stimulus. Superoxide anion-positive leukocytes were assessed by the NBT assay. Cell suspension was incubated in an equal part of NBT solution (1 mg mL^–1^) and added to a slide and later counterstained with the Romanowsky stain-based method (Fast Panoptic Kit, Laborclin, Pinhais, PR, Brazil). The results were the number of NBT-positive cells × 10^6^.^49^

### Statistical Analysis

Results are presented as mean ± SEM of five mice per group per experiment and are representative of two separate experiments. Two-way repeated measures analysis of variance (two-way ANOVA) followed by Tukey’s post hoc test was used to compare groups and doses at all time points when responses were measured at different time points after stimulus injection (mechanical hyperalgesia assessment). One-way ANOVA followed by Tukey’s post hoc test was performed for data from single time point experiments. For histological analyses, the Kruskal–Wallis test was followed by Dunn’s post hoc test. *p* < 0.05 was considered statistically significant. All data analyses were performed using GraphPad Prism 9.0 (GraphPad Software, San Diego, California USA).

## Results

### Rhamnolipid Production

First, RL production was evaluated under submerged liquid cultivation using a basic medium consisting of a salt solution and glycerol as the carbon source. Under these conditions, an RL concentration of 3.5 g/L was achieved after cultivation for 8 days (Table 1). The RL crude extract (1.5 g/L; Table 1) was then obtained through a simple two-step procedure consisting of cold acid precipitation followed by solvent extraction (Figure 1), resulting in a clean RL extract (Table 1 and Figure S1). Furthermore, solvent extractions were carried out in a precipitated RL sample, quite reducing the amount of solvent used and making the whole process more feasible. Consequently, the following purification experiments were run using this easy and clean process for RL production and extraction, with an affordable medium in the very usual and scalable fermentative technology.

**Table 1:** Rhamnolipid Production and Purification: Yields, Composition, and Purity

	rhamnolipid yield (g/L)	dirhamnolipids	Rha_2_C_10_C_10_	recovery rate	purity
rhamnolipids	3.5 ± 0.4^a^				
crude extract	1.5 ± 0.2^a^	95.1%^c^	72.4%^c^		
purified Di-RL	0.7 ± 0.1^b^	97.5%^c^	79.3%^c^	52.3%^d^ ^,^ ^e^	99.0%^f^

a Values plotted are the means of triplicate flasks ± the standard deviation of the mean.

b Values plotted are the means of duplicate ± the standard deviation of the mean.

c Calculated through mass spectrometry (Table 2).

d Percentage of recovery of purified rhamnolipids from crude extract.

e Sequences of solvent washing and elution (procedure “3”, Figure S3).

f Calculated through NMR proton spectra analysis (Figure S4).

**Figure 1:**
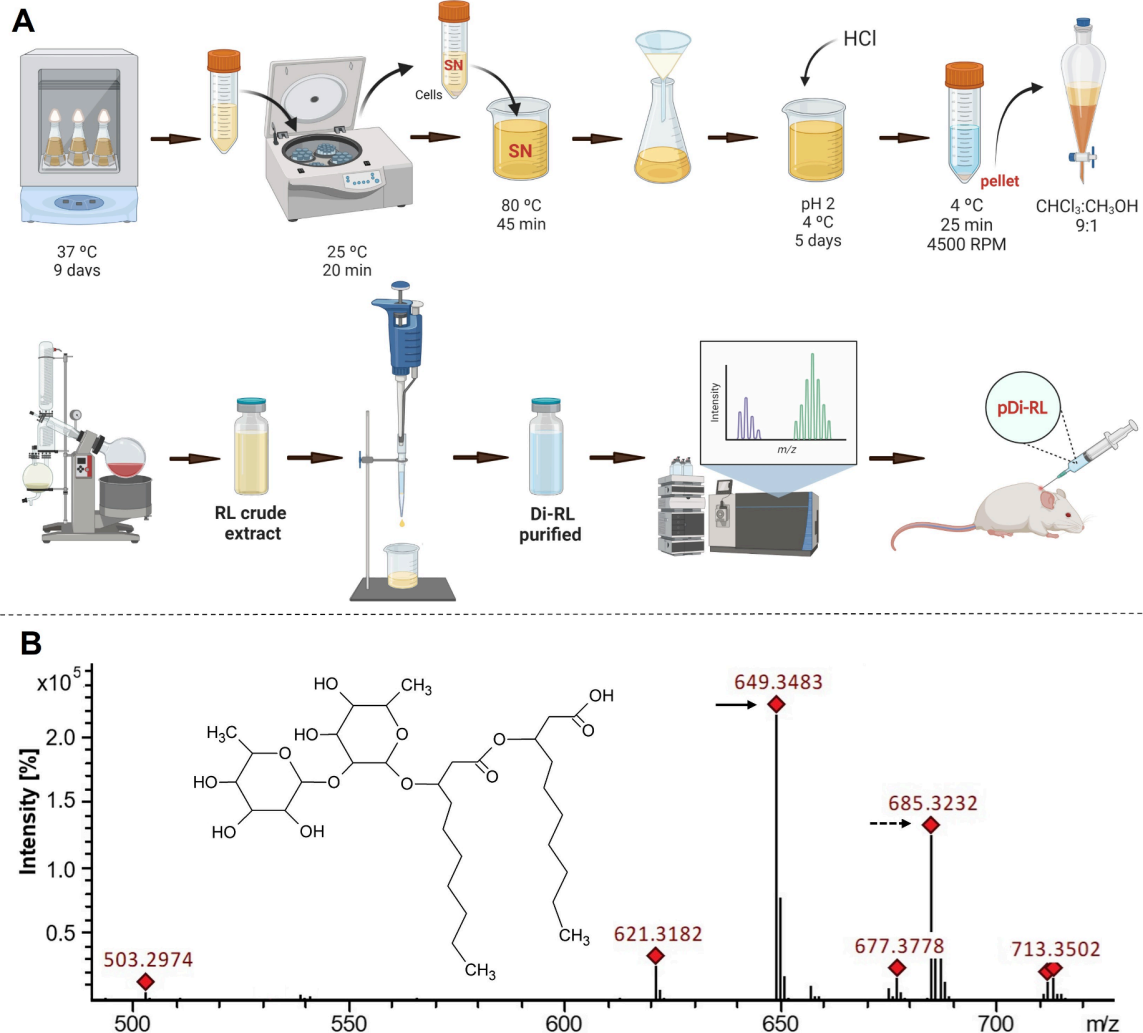
Scheme of dirhamnolipid production and purification (A). Most abundant dirhamnolipid congener (Rha_2_C_10_C_10_) structure and mass spectra of pure dirhamnolipids (B); the solid arrow indicates deprotonated Rha_2_C_10_C_10_, and the dashed arrow indicates the chloride adduct of Rha_2_C_10_C_10_. Created in BioRender.

### Purification of Rhamnolipids

The RL crude extract was submitted to analysis of congener composition by mass spectrometry (Table 2), which showed a very high percentage of Di-RL congeners (over 95%), with the congener Rha_2_C_10_C_10_ (Figure 1) being the most abundant (Table 2). The high percentage of Di-RL congeners also was checked by nuclear magnetic resonance (NMR) through integration of the anomeric proton peaks (Figure S1). The crude extracted was then submitted to the purification procedure (Figure 1) through a homemade silica gel 60 cartridge (Figure S2). Different sequences of solvent washing were run to find the best quality and recovery rate of the Di-RL (Table 1, Figure S3). Different purification procedures were also tried (Table S1), and the silica cartridge was the best one: highly efficient, easy to do, and most cost-effective. A highly pure mixture of RL, 99.0% of RL/97.5% of Di-RL (Tables 1 and 2, Figure S4), was reached following the proposed workflow (Figure 1). Since the pure mixture of RL is mainly composed of Di-RL, hereafter it will be named purified Di-RL (pDi-RL).

**Table 2:** Chemical Composition and Relative Abundance of the Crude Extract and the Purified Rhamnolipid Mixture Produced by *Pseudomonas aeruginosa* PAO1

			crude extract		purified	
	[M-H]^−^	[M-Cl]^−^	congener percentage	total percentage	congener percentage	total percentage
monorhamnolipids				4.9		2.5
Rha_1_C_10_C_8_	475.2657^a^		0.7			
Rha_1_C_8_C_10_						
Rha_1_C_10_C_10_	503.2974		4.2		2.5	
dirhamnolipids				95.1		97.5
Rha_2_C_10_C_8_	621.3225^a^		9.3		7.0	
Rha_2_C_8_C_10_						
Rha_2_C_10_C_10_	649.3483	685.3232	72.4		79.3	
Rha_2_C_10_C_12_	677.3778^a^		6.5		6.8	
Rha_2_C_12_C_10_						
Rha_2_C_10_C_12:1_	675.3584^a^		7.0		4.4	
Rha_2_C_12:1_C_10_						

a Isomers.

### pDi-RL Inhibited Carrageenan-Induced Mechanical Sensitivity

We evaluated the antinociceptive activity of pDi-RL in a model of carrageenan-induced inflammation in the paw using an electronic aesthesiometer. The pDi-RL doses of 0.3 and 3 mg/kg inhibited the sensitivity to mechanical stimulation induced by carrageenan (Figure 2A) after 3 h. We selected the 3 mg/kg dose for the following experiments because it induced more antinociception (70.15% inhibition) when compared to the lowest dose (47.46% inhibition) and induced antinociception at all evaluated time-points (1–5 h). Next, we assessed the mechanical threshold using von Frey filaments. The 3 mg/kg dose induced antinociception in carrageenan-induced inflammation (Figure 2B). We also ruled out any effect induced by only the drug vehicle or pDi-RL (Figure 2B). In particular, the group that received only pDi-RL without carrageenan inflammation showed a similar response to naïve animals, which indicates that pDi-RL does not alter the ability of mice to respond to mechanical stimulation; thus, the diminished response upon carrageenan inflammation represents an antinociceptive effect. Therefore, a 3 mg/kg dose of pDi-RL has an antinociceptive effect on carrageenan-induced inflammatory pain in the animal’s paw, and as expected, the vehicle and pDi-RL alone had no effect.

**Figure 2:**
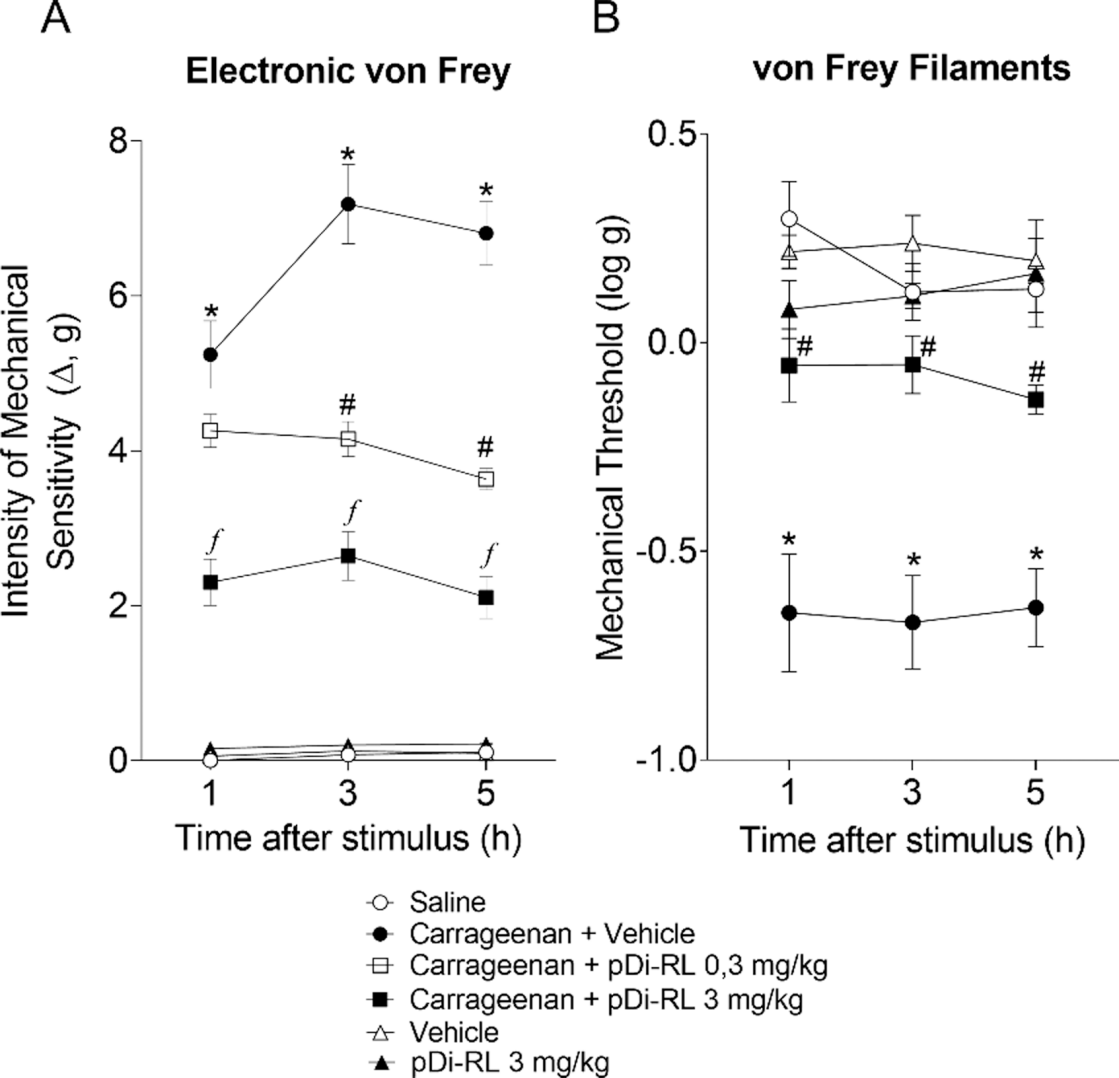
pDi-RL inhibited carrageenan-induced mechanical hyperalgesia. Mice received a subcutaneous (sc) injection of pDi-RL (0.3 and 3 mg/kg) or vehicle (saline 0.9%) 30 min before carrageenan intraplantar (ipl) stimulus (300 μg in 20 μL of saline). The mechanical hyperalgesia was evaluated 1, 3, and 5 h after stimulus by the electronic von Frey method (A) or von Frey filaments (B). Results are presented as means ± SEM of five mice per group per experiment and are representative of two separate experiments. Two-way ANOVA followed by Tukey’s post hoc test was performed. *p < 0.05 carrageenan + vehicle compared to saline group; *#p <* 0.05 pDi-RL (0,3 mg/kg) compared to carrageenan + vehicle group; *^f^p <* 0.05 pDi-RL (3 mg/kg) compared to the other dose and to the carrageenan + vehicle group.

### pDi-RL Inhibited the Spontaneous Nociceptive Behaviors Induced by Acetic Acid and Formalin

To verify the effect of pDi-RL on spontaneous nociceptive behavior, we analyzed the number of abdominal contortions induced by acetic acid and the number of paw flinches induced by formalin. The dose of 3 mg/kg inhibited 52.77% of the writhing response induced by acetic acid over 20 min (Figure 3A). We also found that the tested dose exerted an antinociceptive effect on the formalin-induced paw flinches in both the neurogenic (0–5 min, 48.29%) and inflammatory (10–30 min, 48.81%) phases of the test (Figure 3B). Thus, we demonstrate that pDi-RL, at the tested dose, has antinociceptive effects in these spontaneous nociceptive rodent models.

**Figure 3:**
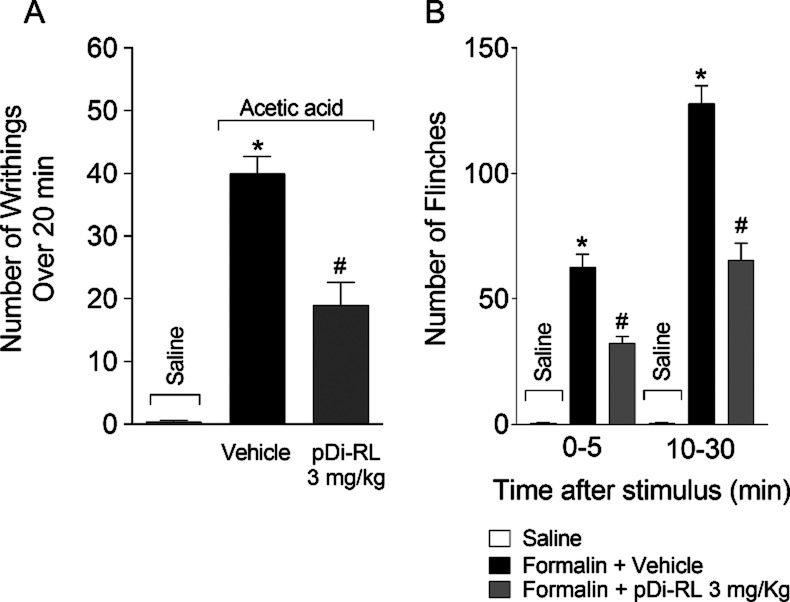
pDi-RL inhibited overt pain-like behavior. Mice were treated subcutaneously (sc) with pDi-RL (3 mg/kg) or vehicle (saline 0.9%) 30 min before intraperitoneal (ip) injection of 0.8% acetic acid (A) and intraplantar (ipl) injection of 1.5% formalin (B). The cumulative number of writhing was evaluated over 20 min, and the total number of paw flinches was evaluated over 30 min. Results are presented as means ± SEM of five mice per group per experiment and are representative of two separate experiments. One-away ANOVA followed by Tukey’s post hoc test was performed. *p < 0.05 carrageenan + vehicle compared to saline group; *#p <* 0.05 pDi-RL (3 mg/kg) compared to carrageenan + vehicle group.

### pDi-RL Inhibited the Leukocyte Infiltrate Induced by Carrageenan Stimulation

To verify the effect of pDi-RL on the leukocyte infiltrate, we performed a histopathology analysis from the cutaneous skin tissue 5 h after carrageenan stimulation. After intraplantar stimulus, there was a significant increase in the leukocyte infiltrate. The treatment with pDi-RL at 3 mg/kg effectively reduced leukocyte infiltrate in the paw induced by carrageenan, as evidenced by histopathological analysis (Figure 4A–D) and quantitative assessment using the ImageJ software (100%, Figure 4E–H). Notably, no significant differences were detected between the saline-treated and pDi-RL-treated groups. These findings suggest that pDi-RL, at a dose of 3 mg/kg, attenuates the inflammatory response by mitigating leukocyte infiltration in the plantar tissue.

**Figure 4:**
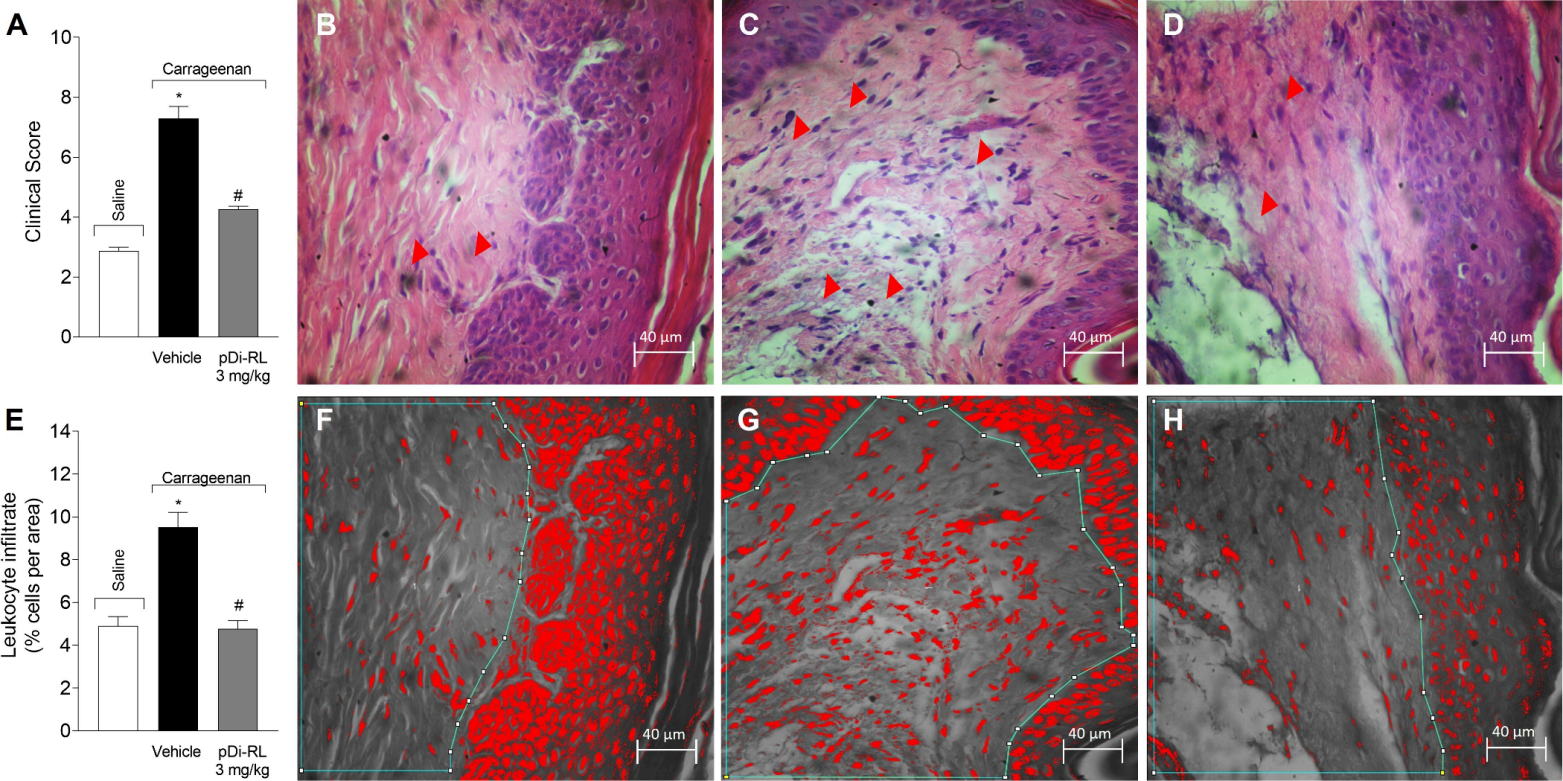
pDi-RL reversed the leukocyte infiltrate caused by carrageenan administration. Mice received subcutaneous (sc) injection of pDi-RL (3 mg/kg) or vehicle (saline 0.9%) 30 min before carrageenan intraplantar stimulus (300 μg, 20 μL, ipl). Five hours after stimulation, the plantar tissue was collected for histological analysis of the leukocyte infiltrate. Panels A–D indicate the histopathological score. The parameters analyzed were vascularization and inflammatory cellular infiltration (arrow). Panels E–H show the total leukocyte recruitment determined using the ImageJ software in % area (highlighted area in green). Results are presented as means ± SEM of five mice per group per experiment. For statistical analysis, the Kruskal–Wallis test was followed by Dunn’s post hoc test. *p < 0.05 carrageenan + vehicle compared to saline group; *#p <* 0.05 pDi-RL (3 mg/kg) compared to carrageenan + vehicle group.

### pDi-RL Inhibited Carrageenan-Induced Total Leukocyte Recruitment, Polymorphonuclear and Mononuclear Cells, and Superoxide Anion Production in the Peritoneal Cavity

To further investigate the activity of pDi-RL in leukocyte recruitment, we used a carrageenan-induced peritonitis model. Five hours after carrageenan intraperitoneal injection, a significant increase occurred in total leukocyte counts as well as in polymorphonuclear and mononuclear cells in the peritoneal exudate. The treatment with pDi-RL at 3 mg/kg reduced the recruitment of total leukocyte (64.13%, Figure 5A) and polymorphonuclear (77.98%, Figure 5B) and mononuclear cells (44.93%, Figure 5C) induced by carrageenan. In addition, NBT-positive cells, as an indication of superoxide anion production, were analyzed in the peritoneal exudate leukocytes. pDi-RL reduced the number of NBT-positive cells recruited to the peritoneal cavity 5 h after carrageenan peritonitis induction (Figure 5D). Thus, pDi-RL at a dose of 3 mg/kg was able to reduce not only the recruitment of leukocytes in the peritoneal exudate but also the production of superoxide anion triggered by carrageenan.

**Figure 5:**
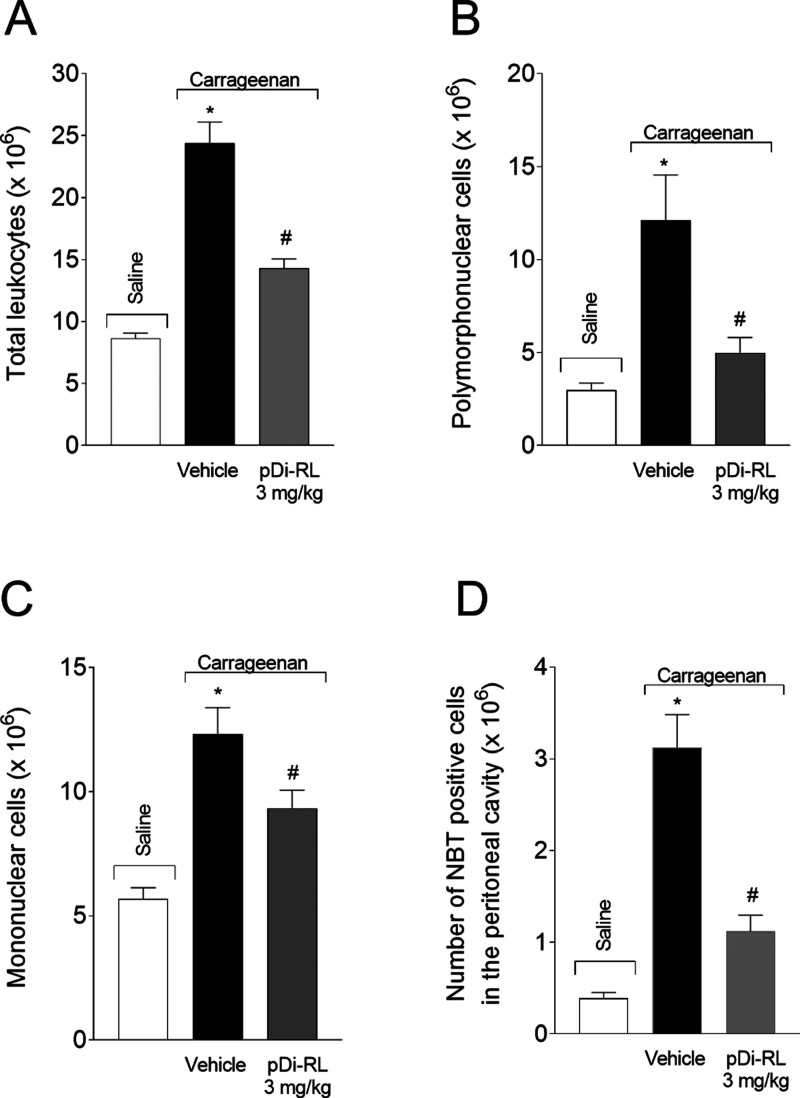
pDi-RL inhibited carrageenan-induced leukocyte recruitment and superoxide anion production in the peritoneal cavity. Mice were treated subcutaneously (sc) with pDi-RL (3 mg/kg) or vehicle (saline 0.9%) 30 min before carrageenan intraperitoneal administration (1 mg, 100 μL, ip). The total number of leukocyte recruitment (A) and polymorphonuclear (B) and mononuclear cells (C) and the number of NBT-positive cells (D) from the peritoneal exudate were evaluated 5 h after the stimulus. Results are presented as means ± SEM of five mice per group per experiment and are representative of two separate experiments. One-way ANOVA followed by Tukey’s post hoc test was performed. *p < 0.05 carrageenan + vehicle compared to saline group; *#p <* 0.05 pDi-RL (3 mg/kg) compared to carrageenan + vehicle group.

## Discussion

RLs are secondary metabolites produced heterogeneously, as a mixture of congeners, mainly by *P. aeruginosa*.^24^ This class of compounds presents a well-known surfactant activity showing great potential for industrial and environmental applications.^50^ RLs have been used in the biodegradation of hydrophobic pollutants, such as in oil spill remediation,^51^ and exhibit superior surface and emulsifying activity compared to conventional chemical surfactants like SDS and Tween 80. Additionally, this biosurfactant demonstrates greater stability under extreme conditions of temperature, pH, and ionic strength relative to SDS and Tween 80.^52^ Herein, we report the production and purification process of Di-RL, along with its antinociceptive and anti-inflammatory activities, as evidenced by the reduced carrageenan-induced pain and inflammation, decreased leukocyte infiltration, and reduced ROS production in recruited leukocytes.

The obtained RL yield (3.5 g/L) was relatively modest. A wide range of RL concentrations have been reported, from 0.43 g/L using palm fatty acid distillate as the sole carbon source under batch fermentation^53^ to 70.56 g/L in soybean oil fed-batch pH stage-controlled fermentation.^54^ Production processes that usually reach expressive yields are those based on complex-composition substrates, mostly the hydrophobic ones^39,55^ and in bioreactor experiments, which might reach concentrations up to 100-fold higher than shake flask experiments.^56^ We have also been working on complex-medium-based processes in which up to 54 g/L of RL can be reached.^55,57^ Even so, the downstream purification processes are usually much more complicated in this type of complex-medium-based cultivation.^38,58^ We were unable to identify an efficient purification procedure for RL in any of these processes (data not shown).

It has been well reported that the most abundant congener of RL is Rha_2_C_10_C_10_; however, the Di-RL abundance presented here (over 95%) is not so frequently observed. Recently, we have described an alternative fermentation technology (static submerged cultivation using membranes of bacterial cellulose as substrate) for RL production that reached 95.6% of Di-RL abundance.^39^ In previous reports claiming high Di-RL-yielding strains, *P. aeruginosa* YM4 ^59^ and *P. aeruginosa* KT1115 ^52^ achieved 85.7 and 88.5% of Di-RL, respectively. Note that here and in our previous report, besides the higher amount of Di-RL attained, we have worked with the PAO1 strain, a worldwide laboratory model of *P. aeruginosa* studies, including for RL production.^56^ Di-RL has been stated to be the more successful one in the treatment of wound healing,^33^ in the stimulation of the immune system of animals and plants,^60–65^ and in scar formation therapy.^34^ Given that, fermentation processes that produce mixtures of RL, highly abundant in Di-RL, might present an industrial advantage.^39,52,59^

Here, we propose a cost-efficient process for the production and purification of a highly pure Di-RL mixture (pDi-RL: RL 99.0%, Di-RL 97.5%). Various purification methods for RL have been described in the literature. In this study, we employed a simpler approach in which the RL crude extract was obtained through a straightforward two-step procedure followed by a single-step purification process. Previous Di-RL purification methods include acid precipitation followed by ethyl acetate extraction, achieving approximately 90% purity.^66^ A more recent report described a four-step procedure (cell and protein removal, acid precipitation, and petroleum ether purification) to reach 89% purity.^67^ In another study, RL was precipitated with ethanol followed by reverse aqueous extraction from ethyl acetate extract and a calcium complexation method, achieving 95.4% purity.^58^ Overall, our production and purification processes demonstrated superior efficiency, simplicity, speed, and cost-effectiveness relative to previously reported methods.

It has already been demonstrated that RLs present cytotoxicity against cervical, leukemic, breast, and bladder cancer cell lines *in vitro*.^29,68^ In addition, the congeners produced in greater proportion here—Di-RL—have therapeutic efficacy in *in vivo* wound healing processes. On the other hand, to our knowledge, the antinociceptive and anti-inflammatory activities have not been described for Di-RL so far. Thus, we evaluated the antinociceptive effect of pDi-RL on mechanical paw hyperalgesia induced by carrageenan. In searching for novel biological effects and potential therapeutic uses for pDi-RL, we applied the acute inflammation induced by carrageenan model, which is a classic model of inflammatory pain with leukocyte recruitment to the inflammatory foci.^69^ Our results show that pDi-RL, administered subcutaneously, significantly reduced the inflamed paw mechanical sensitivity induced by carrageenan between 1 and 5 h at the dose of 3 mg/kg. Furthermore, the antinociceptive effect of this dose was confirmed using methods to assess mechanical hyperalgesia and allodynia. The electronic aesthesiometer is in principle a method to evaluate mechanical hyperalgesia.^41^ This term refers to the sensitization of nociceptive neurons that occurs during inflammation. In mechanical hyperalgesia, there is a nociceptive response that is enhanced upon inflammation. This concept differs from mechanical allodynia, in which there is a response to a stimulus that is not nociceptive per se. To assess this effect, we used the von Frey filaments to which animals do not respond in basal conditions, but there is response upon inflammation characterizing what is described as allodynia.^41^ Both mechanical hyperalgesia and allodynia can be triggered by superoxide anion during inflammation.^70,71^ An interesting point to mention is that the subcutaneous administration of Di-RL in Swiss–Webster mice induced low toxicity at 120 mg/kg per day.^33^ Thus, it is unlikely that the present treatment protocol using 3 mg/kg could induce toxicity. In agreement with that, the sole treatment with pDi-RL did not alter the ability of mice to respond to the electronic aesthesiometer mechanical stimulation, which indicates that pDi-RL did not alter the capability of mice to respond to stimulation.

We further confirmed the pDi-RL antinociceptive activity in two models of overt-like pain behavior: the abdominal writhing model induced by acetic acid and the formalin-triggered paw flinches. pDi-RL inhibited acetic acid induced writhings and both paw flinch phases (neurogenic and inflammatory) induced by formalin. Oxidative stress and free radicals induce overt pain-like behaviors similar to those observed following the injection of acetic acid into the peritoneum and formalin into cutaneous paw tissue.^16^ The writhing response caused by acetic acid and the second phase of the formalin are dependent on superoxide anion since the SOD mimic or superoxide scavenger induces antinociception, respectively.^72,73^ In agreement, the administration of a superoxide anion donor, potassium superoxide, can induce abdominal writhing, paw flinching, and mechanical sensitivity.^16^ Here, we showed that pDi-RL inhibited the writhings induced by acetic acid and the neurogenic and inflammatory phases in formalin-induced paw flinches. Since the neurogenic phase of the formalin test is not dependent on superoxide anion production, this result suggests that pDi-RL may have additional antinociceptive mechanisms such as interfering with the neuronal release of nociceptive molecules. Further studies are needed to assess in deeper detail the antinociceptive mechanisms of pDi-RL not only in inflammation but also in other conditions such as neuropathic pain and cancer pain, which are also dependent on the production of superoxide anion.^74,75^

Using a histopathological approach, we observed that pDi-RL reduced the leukocyte infiltrate induced by carrageenan in the cutaneous tissue. This suggests that its antinociceptive effect may involve the inhibition of leukocyte recruitment to the site of tissue injury. Therefore, we decided to investigate which populations of inflammatory cells had their recruitment affected by pDi-RL. To achieve this aim, we performed a model of peritonitis induced by carrageenan, in which the injection of carrageenan into the peritoneal cavity of mice promotes the generation of ROS.^69^ Carrageenan increased the migration of total leukocytes, differentiated in polymorphonuclear and mononuclear cells, into the peritoneal exudate. Pretreatment with pDi-RL reduced the recruitment of mononuclear cells as well as neutrophils. This reduction of leukocyte recruitment by pDi-RL may be associated with an inhibition of oxidative stress^76^ as evidenced by the decreased levels of superoxide anion in the abdominal cavity. For instance, superoxide anion is essential to the recruitment of neutrophils and their deleterious effects such as tissue damage.^8,9^

Oxidative stress and free radicals induce inflammatory pain. The injection of a superoxide anion donor (potassium superoxide) induces both mechanical and thermal sensitivity, which is inhibited by antioxidants or drugs that interfere with the propagation of oxidative stress. Indeed, the inflammatory pain induced by superoxide anion is accompanied by leukocyte recruitment and oxidative stress.^16,77^ The inflammatory pain caused by carrageenan is dependent on the release of free radicals since treatment with a superoxide dismutase mimetic agent, tempol, inhibits mechanical sensitivity.^78^ In addition, superoxide anion amplifies its own production and triggers the recruitment of leukocytes and pain since tempo and apocynin (a NADPH oxidase inhibitor) diminish its biological effects.^79^ We assessed the superoxide anion production in recruited leukocytes by counting NBT-positive cells in carrageenan-induced peritonitis. We found that pretreatment with pDi-RL reduced the number of NBT-positive cells induced by carrageenan in the peritoneal exudate, suggesting that pDi-RL antinociceptive activity is related to the inhibition of free radical production in recruited leukocytes induced by carrageenan.

It has already been reported that RL presents anti-inflammatory effects by alleviating intestinal injury caused by LPS in broilers,^80^ but our work was the first to demonstrate an antinociceptive activity induced by pDi-RL. In the present study, pDi-RL produced and purified from *Pseudomonas aeruginosa* reduced inflammation and nociceptive behaviors in mice, induced by carrageenan and formalin in the paw and by acetic acid in the peritoneum, as well as presenting inhibitory effects on leukocyte migration and superoxide anion production in carrageenan-induced cutaneous inflammation and peritonitis. The use of RL is safe due to its nontoxicity and high biodegradability, being used in the food, pharmaceutical, petrochemical, and health sectors.^29^ Its biological properties combined with anti-irritant effects and compatibility with the skin allow RL to be used in therapeutic applications.^33^

## Conclusions

To conclude, we demonstrated for the first time the antinociceptive effect of Di-RL in mice and further supported its anti-inflammatory activity. RL was produced by *Pseudomonas aeruginosa* and purified in a fast and simple process. Its novel antinociceptive activity together with previously known activities indicates that this is an important compound to be further explored for industrial application to our benefit in varied fields. We provide a process of production and purification and a novel application adding to the existing literature.

## Supplementary Material



## Data Availability

The data presented in this study are available on reasonable request to the corresponding author.

## Notes

Spectral data (^1^H NMR) with integrated regions of rhamnolipid crude extract; homemade silica gel cartridge; methods for rhamnolipid purification; and relative purity analysis of purified dirhamnolipids (pDi-RL) by proton NMR quantification (PDF)
