# Supplementary material for: Targeted-Produced Dirhamnolipids from Pseudomonas aeruginosa Induce Antinociception in Mice
Source: ACS Omega. 2025 Aug 7;10(32):36056–67. doi: 10.1021/acsomega.5c03648 (PMC12368654; doi:10.1021/acsomega.5c03648)
Supplement: Supplementary file 1 [file ao5c03648_si_001.pdf]

Supplementary Material

Targeted-produced Di-rhamnolipids from *Pseudomonas aeruginosa* induce antinociception in mice.

Kamila B. B. Wessel[1], Ana Paula Mello[3], Ismael Rodrigues Amador[1], Marília F. Manchope[2], Nayara Rampazzo Morelli[2], Anelise Franciosi[2], Tiago H. Zaninelli[2], Mariana M. Bertozzi[3], Cesar A. Tischer[1], Renata M. Martinez[3], Nicole Caldas Pan[1], Marcela M. Baracat[3], Rubia Casagrande[3], Waldiceu A. Verri[2*], Doumit Camilios-Neto[1], Josiane A. Vignoli[1*]

[1]Departamento de Bioquímica e Biotecnologia, Centro de Ciências Exatas, Universidade Estadual de Londrina, 86057-970, Londrina, Brazil.

[2]Departamento de Imunologia, Parasitologia e Patologia Geral, Centro de Ciências Biológicas, Universidade Estadual de Londrina, 86057-970, Londrina, Brazil.

[3]Departamento de Ciências Farmacêuticas, Centro de Ciências da Saúde, Universidade Estadual de Londrina, 86057-970, Londrina, Brazil.

*Correspondence: javignoli@uel.br and waverri@uel.br

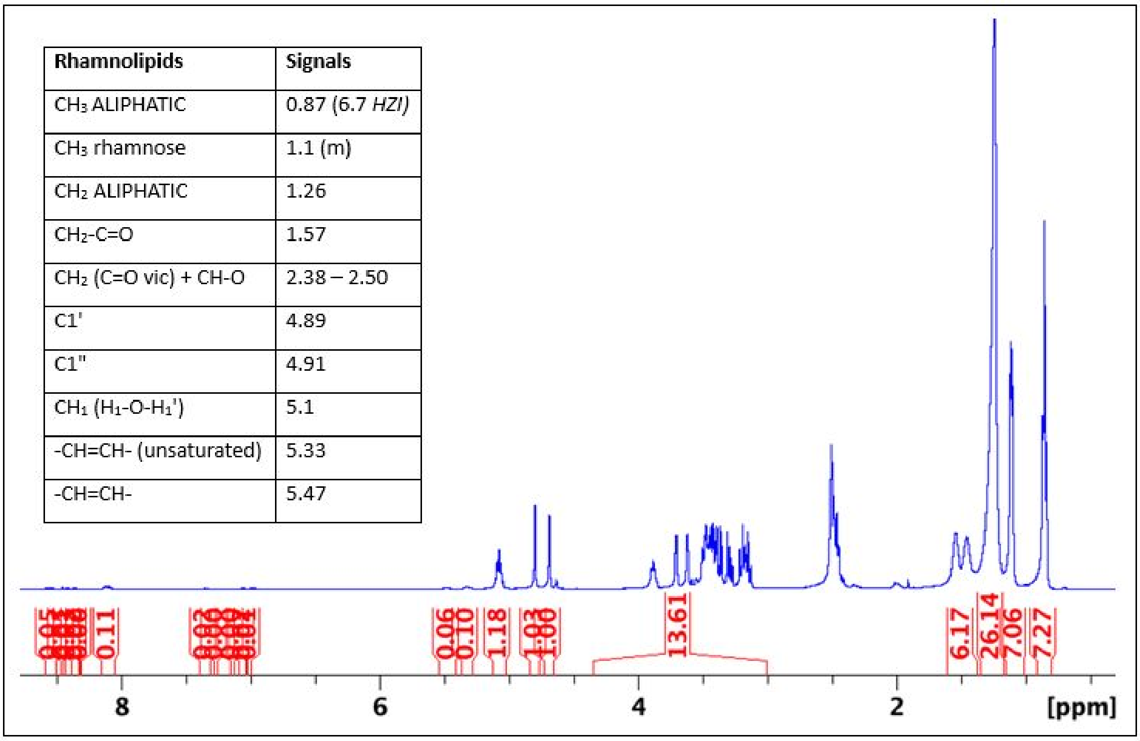

Rhamnolipids	Signals
CH_3 ALIPHATIC	0.87 (6.7 *HZl)*
CH_3 rhamnose	1.1 (m)
CH_2 ALIPHATIC	1.26
CH_2-C=O	1.57
CH_2 (C=O vic) + CH-O	2.38 – 2.50
C1'	4.89
C1''	4.91
CH_1 (H_1-O-H_1')	5.1
-CH=CH- (unsaturated)	5.33
-CH=CH-	5.47

Fig. S1 Nuclear magnetic resonance proton spectra with the integrated regions of rhamnolipids crude-extract

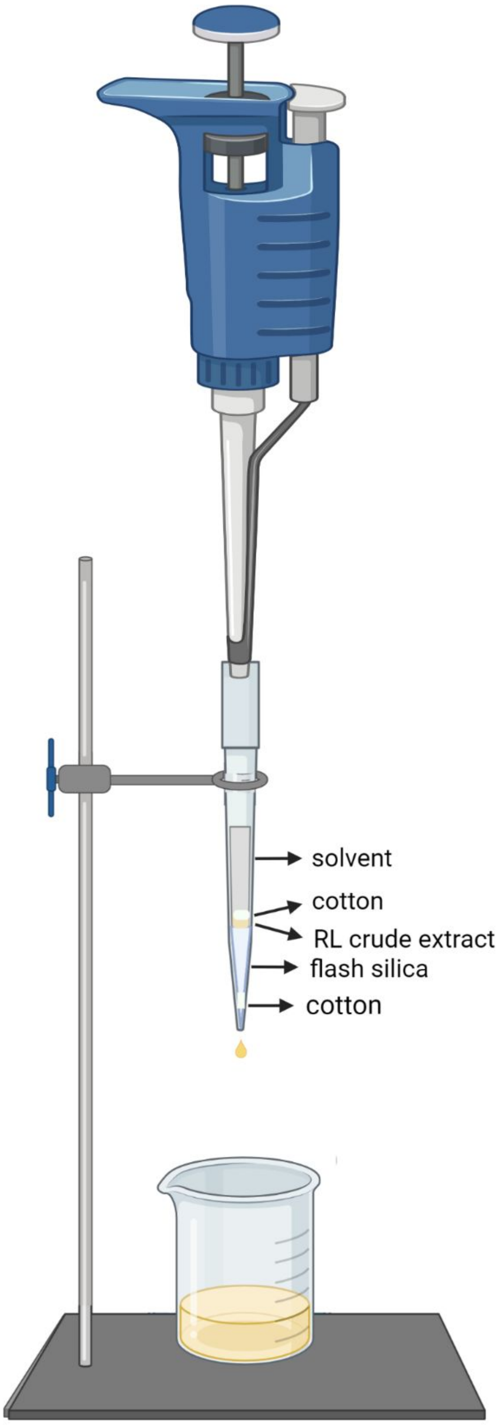

Fig. S2 Home-made silica gel cartridge

Created in BioRender. Wessel, K. (2025) https://BioRender.com/04ffqcl

A

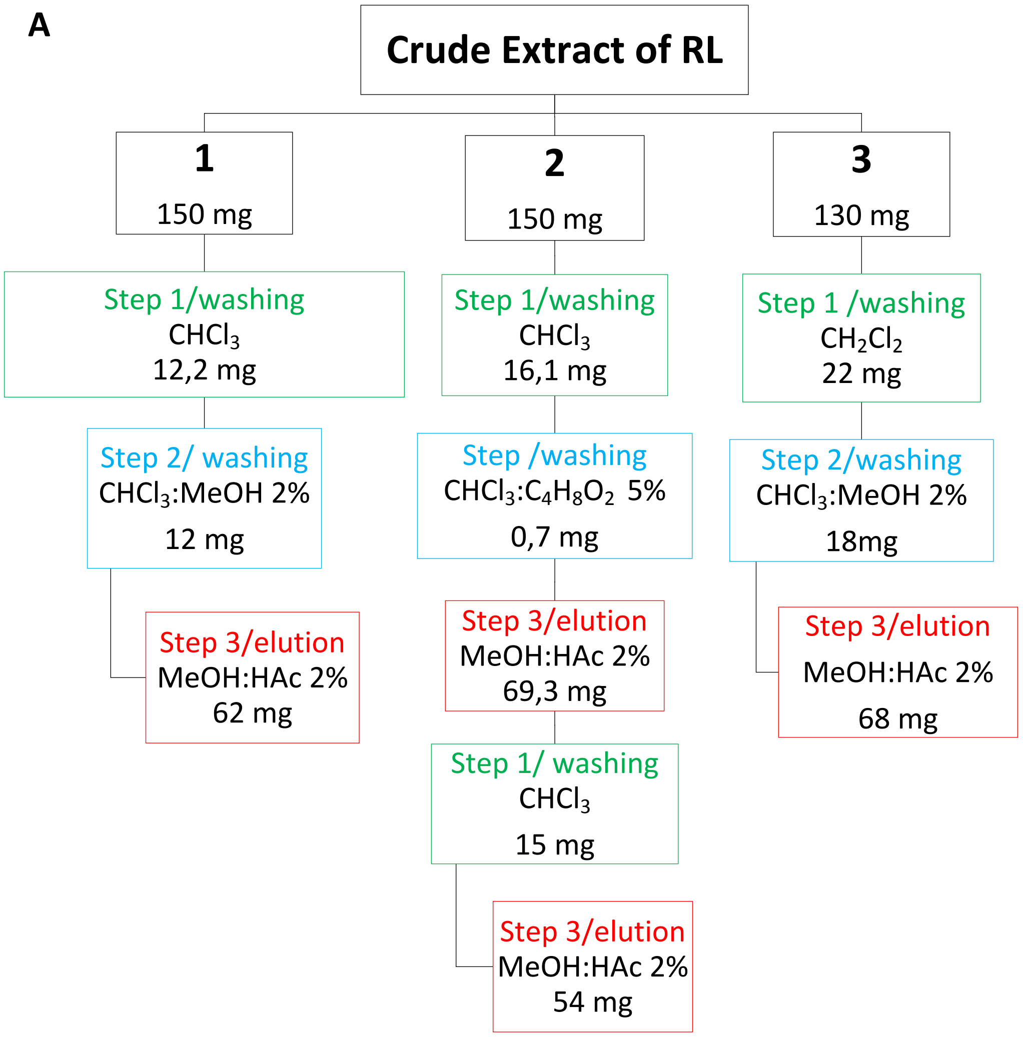

B

Solvent-washing sequences	Recovery Rate
1	41.3 %
2	36.0 %
3	52.3 %

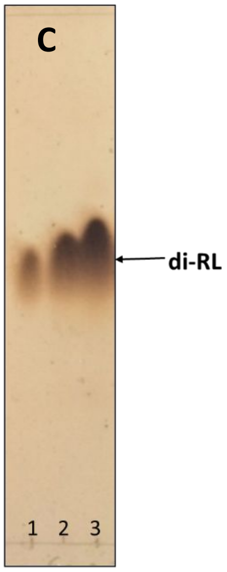

Fig. S3 Evaluation of solvent washing and elution procedure for silica gel cartridge purification.
(A) sequences of solvent-washing, (B) recovery rate, (C) orcinol stained analytical TLC from the recovery di-RL from solvent-washing sequences 1, 2 and 3. TLC mobile phase: $CHCl_3$:CH_3OH (20%):$C_2H_4O_2$ (3%).

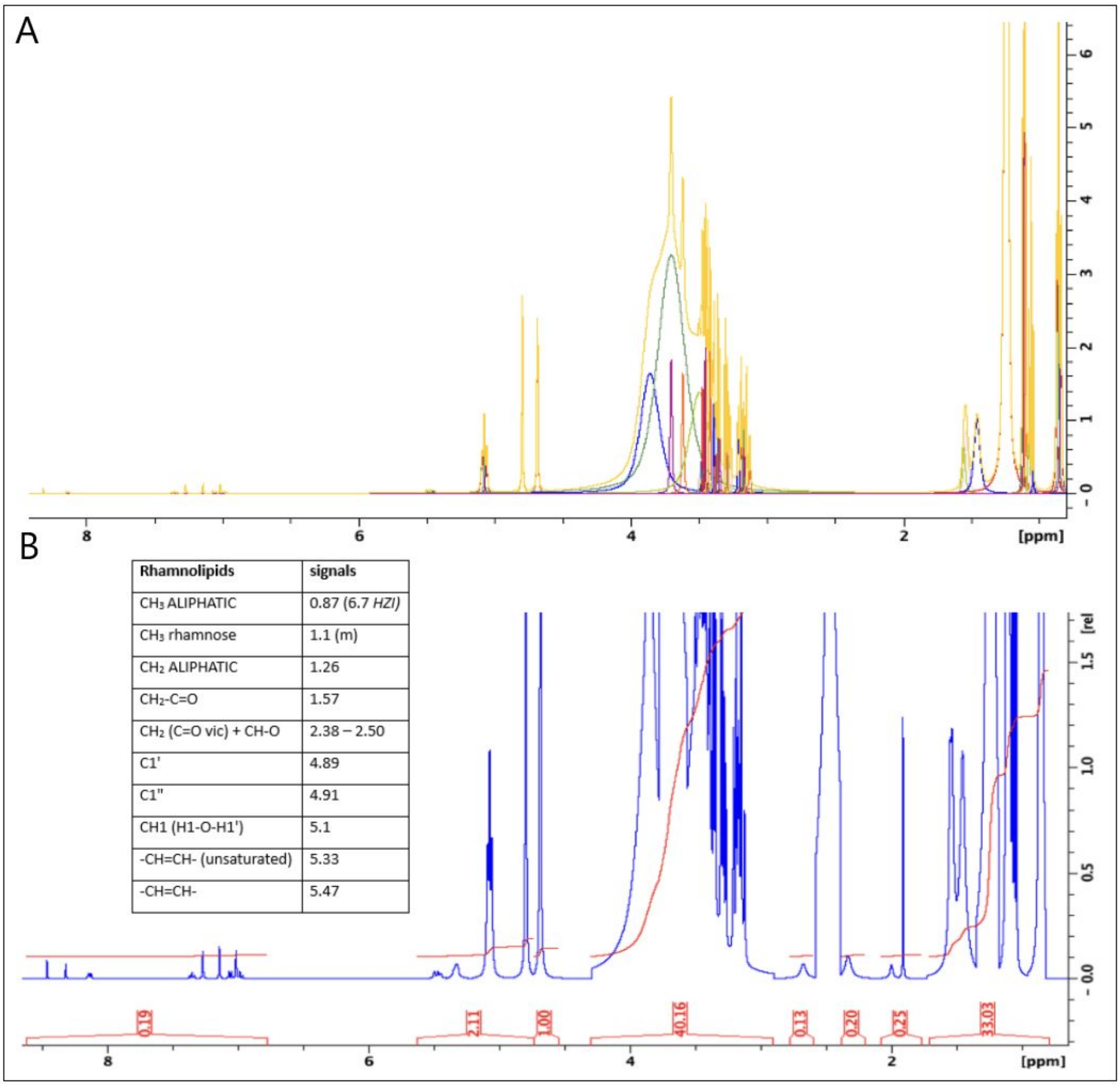

Rhamnolipids	signals
CH_3 ALIPHATIC	0.87 (6.7 *HZI)*
CH_3 rhamnose	1.1 (m)
CH_2 ALIPHATIC	1.26
CH_2-C=O	1.57
CH_2 (C=O vic) + CH-O	2.38 – 2.50
C1'	4.89
C1"	4.91
CH1 (H1-O-H1')	5.1
-CH=CH- (unsaturated)	5.33
-CH=CH-	5.47

Fig S4 Relative purity analysis of purified di-rhamnolipids by nuclear magnetic resonance proton-quantification. (A) Deconvolution sum of each hydrogen signal present in the rhamnolipids nuclear magnetic resonance proton spectra. (B) Calculated area for each signal from the deconvolution of the hydrogen signals in rhamnolipid spectrum, with the integrated regions and values.

Relative purity analysis of purified di-rhamnolipids (pdi-RL) by nuclear magnetic resonance proton-quantification.

The determination of the rhamnolipids purity was carried out by the modified method of relative quantification (Bharti & Roy, 2012). The analysis was performed from the deconvolution graphs of the ^{1}H NMR spectra, obtained in the Bruker Avance III equipment, using pulse program *zgpr* with 8 scans (*ns*), with fid size of 65536 (*td*, *size of fid*), relaxation time (*d1, relaxation delay*) of 1 s, with polynomial automatic adjustment of the baseline. First, the deconvolution of all hydrogen signals was performed except for the solvent DMSO-*d6*, and the graph was reconstructed from the quantitative data Fig S5A. The integrated regions were 20-times larger than the signal width at their average height. For calculation of relative purity, the areas of hydrogen signals (Table 1) were

aggregated as values of the analyte, rA. All other signals present in the spectrum were assigned as impurities, rC. Note that no perceptible signal of these contaminants in the regions quantified as analyte in the 2D spectrum of ^{1}H-^{13}C HSQC (*hsqcedetgpsp*.3, td 512x256, number of scans *ns* 16) (data not shown). The degree of purity was obtained from the ratio of the mass of total hydrogens of the rhamnolipids against the total mass of hydrogens of the sample following the formula below:

$$p\% = \frac{rA}{(rA + rC)} \,.\, 100$$

The relative hydrogen measurement considered that the total mass of hydrogen is representative of the total mass of the sample, and that the hydrogens of each molecular entity end up composing the fraction of the whole. Thus, once all the rhamnolipid hydrogen signals were characterized, the sum of their areas is a representation of their mass present in the sample. Applying the integration data for the main analyte (rhamnolipids), it leads to a percent purity of 99.001%.

Table S1 Evaluation of different methods for rhamnolipids purification

**Purification procedures**	**Solvent volume per mass of crude extract (mL/g)**	**Recovery rate (%)** [a]	**Purification Efficiency** [b]	**Technical difficulties** [c]
**Preparative Thin Liquid Chromatography**	3.5	65.6	Low	High
***Flash Liquid* Chromatography**	2.5	69.0	Medium	High
**Home-Made Silica Cartridge**	0.2	52.3	High	Low

[a] Recovery rate were estimated based on the difference from initial to final masses

[b] Purification efficiency was estimated by analytical TLC performed after each procedure

[c] The evaluation of technical difficulties was based on preparation time, materials used and conditions necessary to perform the technique

Reference

Bharti, S. K., & Roy, R. (2012). Quantitative ^{1}H NMR spectroscopy. *TrAC Trends in Analytical Chemistry*, *35*, 5–26. https://doi.org/10.1016/J.TRAC.2012.02.007
